# Adverse Drug Reactions of Antihypertensives and CYP3A5*3 Polymorphism Among Chronic Kidney Disease Patients

**DOI:** 10.3389/fphar.2022.848804

**Published:** 2022-03-14

**Authors:** Fei Yee Lee, Farida Islahudin, Abdul Halim Abdul Gafor, Hin-Seng Wong, Sunita Bavanandan, Shamin Mohd Saffian, Adyani Md Redzuan, Mohd Makmor-Bakry

**Affiliations:** ^1^ Centre for Quality Management of Medicines, Faculty of Pharmacy, Universiti Kebangsaan Malaysia, Kuala Lumpur, Malaysia; ^2^ Clinical Research Centre, Hospital Selayang, Ministry of Health Malaysia, Batu Caves, Malaysia; ^3^ Nephrology Unit, Department of Medicine, Universiti Kebangsaan Malaysia Medical Centre, Kuala Lumpur, Malaysia; ^4^ Nephrology Department, Hospital Selayang, Ministry of Health Malaysia, Selangor, Malaysia; ^5^ Nephrology Department, Hospital Kuala Lumpur, Ministry of Health Malaysia, Kuala Lumpur, Malaysia

**Keywords:** adverse drug reaction, chronic kidney disease, pharmacogenetics, CYP3A5, antihypertensive drugs

## Abstract

Chronic kidney disease (CKD) patients may be more susceptible to adverse drug reactions (ADRs), given their complex medication regimen and altered physiological state driven by a decline in kidney function. This study aimed to describe the relationship between CYP3A5*3 polymorphism and the ADR of antihypertensive drugs in CKD patients. This retrospective, multi-center, observational cohort study was performed among adult CKD patients with a follow-up period of up to 3 years. ADRs were detected through medical records. CYP3A5*3 genotyping was performed using the direct sequencing method. From the 200 patients recruited in this study, 33 (16.5%) were found to have ADRs related to antihypertensive drugs, with 40 ADRs reported. The most frequent ADR recorded was hyperkalemia (n = 8, 20.0%), followed by bradycardia, hypotension, and dizziness, with 6 cases (15.0%) each. The most common suspected agents were angiotensin II receptor blockers (n = 11, 27.5%), followed by angiotensin-converting enzyme inhibitors (n = 9, 22.5%). The CYP3A5*3 polymorphism was not found to be associated with antihypertensive-related ADR across the genetic models tested, despite adjustment for other possible factors through multiple logistic regression (*p* > 0.05). After adjusting for possible confounding factors, the factors associated with antihypertensive-related ADR were anemia (adjusted odds ratio [aOR] 5.438, 95% confidence interval [CI]: 2.002, 14.288) and poor medication adherence (aOR 3.512, 95% CI: 1.470, 8.388). In conclusion, the CYP3A5*3 polymorphism was not found to be associated with ADRs related to antihypertensives in CKD patients, which requires further verification by larger studies.

## Introduction

An adverse drug reaction (ADR) is defined as a noxious and unintended reaction to a drug at doses normally used in humans (World Health Organization, 2002). ADRs are a burden to the healthcare system, as patients with ADRs are found to have a longer duration of hospitalization and higher hospitalization costs (Suh et al., 2000). However, studies related to ADRs are mainly conducted among hospitalized patients rather than patients seen in outpatient settings (Laville et al., 2020). Among patients with chronic illness, chronic kidney disease (CKD) patients frequently report ADR. CKD patients might be more susceptible to ADRs given the need for multiple therapeutic agents to manage the various comorbidities of CKD (Laville et al., 2020).

ADRs could be potentially difficult to be predicted, given the multifactorial nature of ADRs, especially among CKD patients. Due to different physiological factors as a result of kidney function decline, it is difficult to extrapolate findings on the propensity of ADRs from existing studies among the general population to CKD patients. In addition, the unpredictable interindividual drug responses in the form of ADR might be driven by genetic polymorphisms that affect drug metabolism pathways and drug metabolism activity (Zanger and Schwab, 2013). Genetic polymorphisms that affect the drug metabolism pathways, such as the cytochrome P (CYP) 450 system, are of clinical prominence, as CYP450 metabolizes more than 80% of drugs (Zanger and Schwab, 2013). Therefore, CYP450 pharmacogenomics might be a promising approach to mitigate ADRs, especially in CKD patients.

The CYP3A family is the most abundantly expressed isoform of CYP450 enzymes, especially the CYP3A4 and CYP3A5 enzymes (Dorji et al., 2019). The presence of single-nucleotide polymorphism (SNP) in genes encoding these enzymes may result in variations in expression and activity of these enzymes (Lolodi et al., 2017). The consequential change in CYP enzyme activity causes alterations to the pharmacokinetic properties of the affected drugs, which then causes variation in the drug effects (Dorji et al., 2019). While SNPs to genes-encoding CYP3A4 enzyme are rare in East Asians, SNPs of the *CYP3A5* gene, especially CYP3A5*3 (rs776746), are more common in Asian populations with an estimate of 65.7–71.3% (Dorji et al., 2019; Liang et al., 2021). The CYP3A5*3 polymorphism, in which guanine (G) replaces adenine (A) at position 6,986 of the gene, causes alternative splicing that affects the quantity of the functioning CYP3A5 enzyme, which reduces the metabolic capacities of CYP3A5-substrate drugs (Kuehl et al., 2001; Zhang et al., 2014). The wild-type allele of the *CYP3A5* gene is CYP3A5*1, in which individuals with this allele express the CYP3A5 protein (Kuehl et al., 2001).

*CYP3A5* polymorphism is potentially associated with hypertension and blood pressure regulation (Zhang et al., 2014). Furthermore, drug responses might differ according to the status of CYP3A5*3 polymorphism. The blood pressure response to the angiotensin-converting enzyme inhibitor (ACEI) was previously found to be significantly blunted in CYP3A5*1 carriers, which might be contributed by sodium-retaining effects and/or elevated activity of the renin-angiotensin-aldosterone system (RAAS) (Eap et al., 2007). The opposite might occur in the absence of the CYP3A5*1 allele, in which the adverse effect of hyperkalemia commonly seen with RAAS blockade by angiotensin-converting enzyme inhibitor (ACEI), angiotensin II blocker (ARB), or spironolactone might be potentiated. However, there is limited evidence linking *CYP3A5* polymorphism with these agents. In addition, CKD patients with RAAS blockers might be more predisposed to clinically significant hyperkalemia, especially those with advanced CKD (Weir and Rolfe, 2010).

CYP3A5*3 polymorphism was previously reported to be associated with peripheral edema associated with amlodipine in general population (Liang et al., 2021). However, the generalizability of ADR studies among the general population to the CKD population might be limited, given the possibility of CKD influencing drug disposition (Yeung et al., 2014). In addition to reduction of renal clearance, CKD might attenuate a number of CYP-mediated metabolic pathways through several possible mechanisms, ranging from direct competitive inhibition by uremic constituents to alterations in gene transcription and translation (Yeung et al., 2014).

Hypertension is closely associated with CKD as a decline in kidney function precipitates the increase in blood pressure, but hypertension accelerates the progression of CKD (Judd and Calhoun, 2015). Optimization of antihypertensive therapy is therefore important for CKD patients. However, antihypertensive agents were found to be commonly related to ADRs (Laville et al., 2020). The identification of the potential contributing factors to ADRs related to antihypertensive agents is therefore important for preventive measures to minimize the suboptimal effects associated with antihypertensive agents in CKD patients. This study aimed to describe the relationship between CYP3A5*3 polymorphism and ADR to antihypertensive drugs in CKD patients.

## Materials and Methods

### Study Design and Study Population

This retrospective, multi-center, observational cohort study was performed among adult CKD patients aged 18 years and above with routine care for at least 5 years in nephrology specialist clinics in three Malaysian tertiary hospitals. Patients who were pregnant, lactating, had dementia, had incomplete medication records, or kidney transplant recipients were excluded. Written informed consent was obtained from all patients included in this study.

The study protocol was approved by the Medical Research Ethics Committee, Malaysia (KKM.NIHSEC.P19-2320 (11)) and the Universiti Kebangsaan Malaysia Research Ethic Committee (UKM PPI/111/8/JEP-2020-048). This study was conducted in compliance with the Declaration of Helsinki and Malaysian Good Clinical Practice Guidelines.

### Data Collection

Each participant who provided informed consent was assigned a unique subject identification number linked to a password-protected database. Information about all medications used, use of traditional/complementary medicine (TCM), and adherence to medications was retrieved from the medical records. The name, dosage form, dose, frequency, timing of administration, and duration of administration of each medication were recorded. Medications used were categorized in accordance with the World Health Organization Anatomical Therapeutic Classification system classification (WHO Collaborating Centre for Drug Statistics Methodology, 2019). Consumption of traditional/complementary medicine was defined as the use of herbs (or botanicals) or over-the-counter nutritional/dietary supplements which were not prescribed by hospitals or health clinics, based on patient recall (Lee et al., 2021b).

Patients’ medical records were then accessed to obtain sociodemographic characteristics, clinical information, laboratory data, medication records, as well as ADRs related to antihypertensives that were reported and occurred during the study period. ADRs were then assessed using the Naranjo scale, whereby ADRs with a causality probability category score equivalent to the “possible” category of at least a score of 1 and above were included (Naranjo et al., 1981; Laville et al., 2020). For reproducibility, the causality assessment for each ADR was performed by two pharmacists independently. The assessment also included a review of all concurrent drugs and TCM during the ADRs to detect any potential drug–drug or TCM–drug interactions. In view of the various herbal concoctions used in TCM, the identification of the active ingredients of each TCM was performed using the QUEST3+ System of the National Pharmaceutical Regulatory Agency, a centralized online system for product registration and licensing in Malaysia. The ADRs were grouped according to the Medical Dictionary for Regulatory Activities (MedDRA).

The kidney function of patients was estimated using the Chronic Kidney Disease Epidemiology Collaboration equation. Stages of CKD and proteinuria status of the patients were categorized as per Kidney Disease Improving Global Outcomes (KDIGO) 2012 guidelines (KDIGO, 2012).

Medication adherence was assessed through medical records. Medication adherence was considered poor if discrepancies from prescribers’ orders for drug, dose, frequency, and duration in any of the three medication adherence phases were recorded (Vrijens et al., 2012; Lee et al., 2021b).

### Sample Size Calculation

Calculation of sample size was performed using G*Power 3.1.9.7 for logistic regression, with *α* = 0.05, 1-β = 0.8, Pr(Y = 1|X = 1) H0 of 0.211 based on 21.1% of ADRs reported among those without CYP3A5*3/*3 genotype (Liang et al., 2021); Pr(Y = 1|X = 1) H1 of 0.317 based on 31.7% of ADRs reported in those with CYP3A5*3/*3 genotype (Liang et al., 2021); and hence the effect size based on the odds ratio of 1.74 derived from the software. Based on the calculation, 166 patients were required.

### Detection of CYP3A5*3 Gene Polymorphism

Venous blood was collected from patients and DNA was extracted using the DNeasy^®^ Blood and Tissue extraction kit (Qiagen, Hilden, Germany). A polymerase chain reaction of the region encompassing the CYP3A5*3 gene polymorphism was performed using the TopTaq Mastermix Kit (Qiagen, Hilden, Germany), followed by direct sequencing using the BigDye^®^ Terminator version 3.1 cycle sequencing kit, which was run on a 96-capillary 3730xl DNA Analyzer (developed by Applied Biosystem, United States and produced by Thermo Fisher Scientific) (Boutin et al., 2000; Lee et al., 2021a). Other gene polymorphisms of the *CYP3A5* gene were not assessed, given the low prevalence of other polymorphisms in South East and East Asian populations (Dorji et al., 2019).

### Statistical Analysis

The results are presented as frequencies and percentages for categorical data. Numerical data are presented as median (interquartile range, IQR), as the numerical data were found to be non-normally distributed upon inspection of histograms. Pearson’s Chi-square test for independence was used to study the association between categorical data, but if the assumptions of the test were not met, Fisher’s exact test was used instead. The Mann–Whitney test was used for the non-normally distributed numerical data. A *p*-value < 0.05 was considered statistically significant.

Adherence to the Hardy–Weinberg equilibrium assumption was examined using a Chi-square test which compared the study results with the predicted allele and genotype distribution derived from the Hardy–Weinberg equation. A *p*-value > 0.05 indicated that the observed genotype distribution was consistent with the assumptions of Hardy–Weinberg Equilibrium (Tahir et al., 2020). The association of genetic polymorphism with ADRs related to antihypertensives was then assessed using logistic regression on the genetic models of dominant [0 = CYP3A5*1/*1 (TT), 1 = CYP3A5*1/*3 (TC) + CYP3A5*3/*3 (CC)], recessive [0 = CYP3A5*1/*1 (TT) + CYP3A5*1/*3 (TC), 1 = CYP3A5*3/*3 (CC)], additive [0 = CYP3A5*1/*1 (TT), 1 = CYP3A5*1/*3 (TC); 2 = CYP3A5*3/*3 (CC)], and allele models [0 = CYP3A5*1 (T), 1 = CYP3A5*3 (C)] (Yoshida et al., 2009).

Simple and multiple stepwise logistic regression were performed on all variables, with variables of *p*-value < 0.25 found from the simple logistic regression included in the multiple logistic regression (Hosmer et al., 2013). Factors with a *p*-value < 0.05 in the multiple logistic regression were considered significant. The possibility of multicollinearity among variables was examined (Meyers et al., 2006; Hosmer et al., 2013). The resulting model was also checked for interaction terms to be adjusted if any were found. The Hosmer–Lemeshow goodness-of-fit test, classification tables, and area under the receiver operating characteristic curve were used to investigate any misrepresentation of data (Hosmer et al., 2013). All statistics were performed using the IBM Statistical Package for Social Science for Windows version 23 (IBM Corp, Armonk, NY, United States).

## Results

### Study Population

Two hundred patients were recruited in this study, with half of them (n = 100, 50.0%) being females, and a median age of 58.5 years (IQR 26.0 years). The study patients had a median of 6 medications (range: 2–15) at baseline. Out of the 200 patients, 33 (16.5%) were found to have ADRs related to antihypertensive drugs, in which most of them were female (n = 23, 69.7%) and approximately half (n = 16, 48.5%) had a baseline eGFR of less than 30 ml/min/1.73 m^2^. The CYP3A5*3 allele frequency was found to be 54.3% (n = 217 out of 400, as one person had two *CYP3A5* alleles). The distribution of the genotypes fulfilled the Hardy–Weinberg equilibrium assumptions (*p* = 0.968). The CYP3A5*1/*1 genotype was found in 42 (21.0%) patients, while the CYP3A5*1/*3 genotype was found in 99 (49.5%) patients, and the CYP3A5*3/*3 genotype was found in 59 (29.5%) patients (Table 1).

**TABLE 1:** Demographic characteristics and factors associated with antihypertensive-related ADR (simple logistic regression).

Variable	Number of patients without ADR, *n* (%) (*n* = 167)	Number of patients with ADR, *n* (%) (*n* = 33)	*p*-value^a^	Odds Ratio (95% CI)	*p*-value
Female gender	77 (46.1)	23 (69.7)	0.021	2.688 (1.205, 5.997)	0.016
Ethnicity					
Malay	83 (49.7)	20 (60.6)		1.000	
Chinese	67 (40.1)	9 (27.3)	0.383^c^	0.557 (0.238, 1.304)	0.178
Others	17 (10.2)	4 (12.1)		0.976 (0.296, 3.221)	0.969
Age, median (IQR)	59.0 (25.0)	55.0 (33.3)	0.215^b^	0.982 (0.959, 1.005)	0.118
Baseline eGFR <30	62 (37.1)	16 (48.5)	0.245	1.594 (0.752, 3.379)	0.224
A3 Albuminuria	65 (43.0)	16 (48.5)	0.215	1.764 (0.781, 3.985)	0.172
Cause of CKD					
Glomerulonephritis	25 (15.0)	4 (12.1)		1.000	
Hypertension	17 (10.2)	3 (9.1)		1.103 (0.219, 5.567)	0.906
Diabetes mellitus	51 (30.5)	8 (24.2)	0.646	0.980 (0.269, 3.569)	0.976
Lupus nephritis	30 (18.0)	10 (30.3)		2.083 (0.582, 7.457)	0.259
Others	44 (26.3)	8 (24.2)		1.136 (0.311, 4.156)	0.847
Smoking	8 (4.8)	2 (6.1)	0.671^c^	1.282 (0.260, 6.329)	0.760
Presence of obesity	46 (13.8)	8 (24.2)	0.845	0.864 (0.278, 2.685)	0.800
Presence of anaemia	80 (47.9)	27 (81.8)	<0.001	4.894 (1.921, 12.469)	0.001
Absence of diabetes mellitus	89 (53.3)	23 (69.7)	0.088	2.016 (0.904, 4.496)	0.087
Presence of hypertension	136 (81.4)	23 (69.7)	0.156	0.524 (0.227, 1.213)	0.131
Presence of dyslipidemia	122 (73.1)	20 (60.6)	0.207	0.567 (0.261, 1.235)	0.153
Presence of congestive cardiac failure	13 (7.8)	2 (6.1)	1.000^c^	0.764 (0.164, 3.557)	0.732
Presence of gout	36 (21.6)	8 (24.2)	0.818	1.164 (0.484, 2.800)	0.734
Number of medications at baseline, median (IQR)	7 (4)	6 (5)	0.876	1.028 (0.891, 1.185)	0.707
Traditional/complementary medicine use	21 (12.6)	3 (9.1)	0.772^c^	0.695 (0.195, 2.480)	0.575
Poor medication adherence	51 (30.5)	18 (54.5)	0.010	2.729 (1.276, 5.838)	0.010
ACEI/ARB use	124 (74.3)	23 (69.7)	0.666	0.798 (0.351, 1.810)	0.589
Beta blocker use	76 (45.5)	16 (48.5)	0.849	1.127 (0.534, 2.380)	0.754
Calcium channel blocker use	112 (67.1)	25 (75.8)	0.414	1.535 (0.650, 3.623)	0.329
Diuretic use	55 (32.9)	12 (36.4)	0.840	1.164 (0.534, 2.536)	0.703
Spironolactone use	17 (10.2)	7 (21.2)	0.074^c^	2.376 (0.897, 6.290)	0.082
Alpha-blocker use	26 (15.6)	7 (21.2)	0.444	1.460 (0.574, 3.714)	0.427
Additive model					
CYP3A5*1/*1	36 (21.6)	6 (18.2)		1.000	
CYP3A5*1/*3	85 (50.9)	14 (42.4)	0.431	0.988 (0.352, 2.776)	0.982
CYP3A5*3/*3	46 (27.5)	13 (39.4)		1.696 (0.587, 4.900)	0.329
Recessive model					
CYP3A5*1/*1+CYP3A5*1/*3	121 (72.5)	20 (60.6)	0.210	1.000	0.176
CYP3A5*3/*3	46 (27.5)	13 (39.4)		1.710 (0.787, 3.717)	
Dominant model					
CYP3A5*1/*1	36 (21.6)	6 (18.2)	0.816	1.000	0.664
CYP3A5*1/*3+ CYP3A5*3/*3	131 (78.4)	27 (81.8)		1.237 (0.474, 3.225)	
Allele model					
CYP3A5*1	157 (47.0)	26 (39.4)	0.281	1.000	0.258
CYP3A5*3	177 (53.0)	40 (60.6)		1.365 (0.796, 2.338)	

a Chi-square tests were carried out unless specified.

b Mann–Whitney test was performed.

c Fisher’s exact test was performed.

ACEI, angiotensin-converting enzyme inhibitor; ADR, adverse drug reaction; ARB, angiotensin II receptor blocker; CI, confidence interval; CYP, cytochrome P450; eGFR, estimated glomerular filtration rate; IQR, interquartile range.

### Description of ADRs Related to Antihypertensives

Forty ADRs related to antihypertensives were reported among the 33 patients, with 7 (21.2%) patients reporting more than one ADR during the study period. The most frequent ADR recorded was hyperkalemia (n = 8, 20.0%), followed by bradycardia, hypotension, and dizziness with 6 cases (15.0%) each (Table 2).

**TABLE 2:** Details of antihypertensive-related ADRs reported.

Type of ADR	Number of ADR, n (%)	Suspected agents (n)	Genotype (n)			Allele (n)	
			CYP3A5 *1/*1	CYP3A5 *1/*3	CYP3A5 *3/*3	CYP3A5*1	CYP3A5*3
Hyperkalemia	8 (20.0)	Perindopril (5), valsartan (1), telmisartan (1), spironolactone (1)	2	3	3	7	9
Bradycardia	6 (15.0)	Atenolol (4), metoprolol (2)	2	2	2	6	6
Hypotension	6 (15.0)	Amlodipine (2), felodipine (1), bisoprolol (1), valsartan (1), telmisartan/amlodipine/metoprolol (1)	1	1	4	3	9
Dizziness	6 (15.0)	Amlodipine (3), losartan (1), telmisartan (1), prazosin (1)	0	4	2	4	8
Acute kidney injury	4 (10.0)	Telmisartan (2), losartan (1), perindopril (1)	1	3	0	5	3
Drug intolerance	4 (10.0)	Prazosin (2), losartan (1), spironolactone (1)	0	2	2	2	6
Blood creatinine increased	3 (7.5)	Perindopril (1), hydrochlorothiazide (1), losartan (1)	1	2	0	4	2
Dry cough	2 (5.0)	Perindopril (2)	0	1	1	1	3
Pedal edema	1 (2.5)	Minoxidil (1)	0	0	1	0	2
Total	40		7	18	15	32	48

ACEI, angiotensin-converting enzyme inhibitor; ADR, adverse drug reaction; ARB, angiotensin II receptor blocker.

The most common suspected agents were ARBs (n = 11, 27.5%), followed by ACEI (n = 9, 22.5%) and calcium channel blockers (n = 7, 17.5%). Most ADRs had one suspected agent implicated per ADR, with only 1 (2.4%) ADR recorded with three suspected agents (Table 2). The number of ADRs by drug class (Table 2) reflected the frequency of prescribed drug classes (ACEI/ARB followed by calcium channel blockers, Table 1). However, the proportion of patients with ADRs varied by pharmacological classes. Most patients were prescribed ACEI/ARB during the study period (n = 147, 73.5%), with 8.2–16.1% ADRs reported among the patients prescribed with the agents, respectively (Supplementary Material). In contrast, among the patients prescribed with calcium channel blockers (n = 137, 68.5%), less ADRs (3.1–6.3%) were reported (Supplementary Material).

As for the causality of the ADRs, most ADRs were classified as “possible” (n = 38, 95.0%), while 2 (5.0%) were categorized as “probable,” respectively. Three (7.5%) ADRs were related with hospitalizations, with no fatality recorded.

One-third of the patients who reported the use of TCM did not specify the name of the TCM used (n = 8). No drug–drug interactions or drug–TCM interactions were detected pertaining to the use of CYP3A5 inducers/inhibitors in the ADRs reported.

About half of the ADRs were managed by discontinuation of the suspected agents (n = 23, 57.5%). Meanwhile, 11 (27.5%) ADRs were addressed by substitution with another agent, and 1 (2.5%) was given correction therapy on top of drug discontinuation. On the other hand, 2 (5.0%) ADRs were managed by dose reduction, while the remaining 1 (2.5%) ADR was managed by reduction in frequency.

### CYP3A5*3 Polymorphism Status and Association With Antihypertensive-Related ADRs

A simple (Table 1) and multiple logistic regression (Table 3) model were performed on variables to identify factors of antihypertensive-related ADRs. From the simple logistic regression, female, anemia, and poor medication adherence were found to be associated with ADRs related with antihypertensives in the study population (Table 1). Variables from the simple logistic regression with a *p*-value of <0.25 were then included into the multiple logistic regression model (gender, ethnicity, age, baseline eGFR, anemia, diabetes mellitus, hypertension, dyslipidemia, poor medication adherence, use of spironolactone, and the recessive model). After adjusting for possible confounding factors, the factors associated with antihypertensive-related ADR were anemia (adjusted odds ratio [aOR] 5.348, 95% confidence interval [CI]: 2.002, 14.288) and poor medication adherence (aOR 3.512, 95% CI: 1.470, 8.388) (Table 3). Additional analysis was performed to determine the relationship between ADRs potentially related to CYP3A5 pharmacokinetics/drug level and CYP3A5*3 polymorphism status, but no significant association was found (*p* = 0.955).

**TABLE 3:** Factors associated with antihypertensive-related ADR (multiple logistic regression).

Variable	b	Adjusted odds ratio (95%CI)	*p*-value
Anemia	1.677	5.348 (2.002, 14.288)	0.001
Poor medication adherence	1.256	3.512 (1.470, 8.388)	0.005

Multiple stepwise logistic regression was performed with adjustment of gender, ethnicity, age, baseline eGFR, albuminuria, diabetes mellitus, hypertension, dyslipidemia, spironolactone use, and recessive model of CYP3A5*3 polymorphism. Multicollinearity and interaction term were not found. Model fit was examined using the Hosmer–Lemeshow test (*p* = 0.995), classification table (84.4%), and area under receiver operating characteristic curve (73.5%).

ADR, adverse drug reaction; CI, confidence interval; CYP, cytochrome P450; eGFR, estimated glomerular filtration rate.

## Discussion

This study provides a novel, pharmacogenomics-driven approach to assess ADRs related to antihypertensives in CKD. CKD patients might be more susceptible to ADRs given the need for multiple medications, in addition to physiological differences contributed by kidney function decline. In addition, the possibility of genetic susceptibility to ADRs has to be taken into consideration. The study findings improved the understanding of the relationship between genetic polymorphism and the development of ADRs related to antihypertensive agents in CKD patients.

In view of the common occurrence of CYP3A5*3 polymorphism among Southeast Asian populations, it is important to understand the association of the gene polymorphism with antihypertensives, which are commonly used in CKD populations and play an important role in managing CKD (KDIGO, 2012). Furthermore, CKD patients have different physiological states given the changes to their excretory functions in contrast with healthy patients, which might affect the propensity of ADRs among these patients. The differences between the general population and CKD patients reflected the limited applicability of the current literature to CKD patients.

ADRs with antihypertensives were found in 16.5% of patients, which was similar to the findings from previous studies of hospitalized cohorts of 10–20% (Danial et al., 2018; Laville et al., 2020). From our study findings, RAAS blockers were found to be the most common suspected agents, which was also similar to that previously reported (Laville et al., 2020). The study findings corresponded with the prescribing pattern of the antihypertensives in the study population, with more than half reported to use ACEI or ARB, corresponding to the RAAS blockade as an important foundation of pharmacotherapy in CKD (KDIGO, 2012). Of note, calcium channel blockers, another frequently implicated agent, were also commonly used among the study cohort. Contrary to the findings of Laville et al. in which acute kidney injury was most commonly reported across several antihypertensive classes (Laville et al., 2020), the present study recorded hyperkalemia as the most common ADR.

The current work demonstrated that anemia was a factor of antihypertensive-related ADR, which concurred with previous reports in CKD patients (Laville et al., 2020). While causality could not be established from this study, the mechanism linking anemia and antihypertensive-related ADR could be explored in future studies. Blood pressure control is likely suboptimal in CKD patients with fluid overload, requiring the use of antihypertensives. A potential mechanism to be examined is pharmacokinetic changes driven by increased fluid status in CKD patients that may have led to a diluted hemoglobin concentration due to volume overload, as supported by the findings that more than half of anemic CKD patients were found to have volume overload (Hung et al., 2015), which led to dilutional anemia (Hildegard Stancu et al., 2018). Nevertheless, the findings improved the understanding of the potential presentation of CKD patients possibly affected by ADRs, in which these patients might have other underlying concurrent medical issues that need to be corrected.

Poor medication adherence was found to be associated with antihypertensive-related ADR in CKD patients, which corroborates the outcomes from previous studies (Laville et al., 2020; Seng et al., 2020). While poor adherence might be multifactorial, the association between poor medication adherence and antihypertensive-related ADR might reflect underlying issues with medication use (Laville et al., 2020). Of these, the most obvious link between ADR and adherence has been reported in studies that document poor adherence to medications that were suspected to be associated with previous ADR episodes (McKillop and Joy, 2013). It is quite possible that the uncomfortable nature of certain ADRs may lead patients to forgo their medication. Given the dynamic nature of medication adherence that might change over time (Unni et al., 2015), this study highlighted the importance of assessing medication adherence regularly among CKD patients through optimization of pharmaceutical care. In addition, current findings that many antihypertensive regimens were adjusted post-ADRs also reflect the need for closer monitoring among these patients, given the close association of antihypertensive drug adjustments with poorer disease outcomes (Lee et al., 2021b).

Although there was a lack of association between CYP3A5*3 and ADR occurrence, further work is recommended. A challenge of studying CYP3A5*3 polymorphism is the potential compensatory functions by CYP3A4 enzyme, owing to the structural similarity with the CYP3A5 enzyme (Lolodi et al., 2017). The low activity of the CYP3A5 enzyme, as a result of CYP3A5*3 polymorphism, might be compensated by the CYP3A4 enzyme, which conceals the phenotype normally expected of the CYP3A5*3 polymorphism. Hence, future work should take CYP3A4 activity into account to evaluate the relationship between CYP3A5*3 polymorphism and susceptibility to ADRs related to antihypertensives in CKD.

Another challenge in utilizing pharmacogenetics in clinical practice is the potential confounding factor of TCM use. TCM may affect the drugs’ metabolism and the propensity of ADRs. An example of this is the use of CYP3A4/5 inhibitors, such as *Allium sativum*, which could potentially inhibit CYP3A5 activity, regardless of the polymorphism status. Of note, TCM use was not found to be a factor of ADRs from our findings. While TCM use is in an increasing trend in this country (Siti et al., 2009; Abdullah et al., 2018), the low number of TCM reported among our study population may reflect underreporting, which limits the ability to detect an association between TCM use and antihypertensive-associated ADRs.

This study’s major strength is the ascertainment of ADR cases from intensive medical record review, which addressed the possible limitation of underreporting found in many ADR studies using data from spontaneous pharmacovigilance reporting systems. In addition, CYP3A5*3 genotyping was performed using the gold standard direct sequencing method. However, a few limitations were noted due to the retrospective study design, which could not imply causality. As such, some ADRs, such as dizziness, might be caused by anemia rather than the implied drugs. On the other hand, there is a possibility of underestimation in the ADRs related to antihypertensives, as some ADRs might not be recorded in the medical records. The small sample size of the study cohort is another limitation in which the study might be underpowered to detect the association between CYP3A5*3 polymorphism and specific antihypertensives or specific doses. In addition, the retrospective nature of the study renders limitations in detailed analysis pertaining to drug–TCM interactions. It was challenging to verify the detailed composition of each TCM, not only due to incomplete data but also due to the fact that many TCMs are not registered with the Ministry of Health, which renders the complete contents of unregistered TCMs unknown (Lee et al., 2020).

In conclusion, the findings of the study indicate that ADRs related with antihypertensives among CKD patients were not associated with CYP3A5*3 polymorphism alone among our study cohort, which requires verification through further studies with greater sample size. These ADRs might be propagated by pathways other than CYP3A5-related metabolism. Additional prospective studies with multiple genetic polymorphisms, and assessments of fluid status could be conducted to verify the findings.

## Data Availability

The data analyzed in this study are subject to the following licenses/restrictions: The authors do not have permission to share the data. The data underlying the results presented in the study are available upon request from the corresponding author for researchers who meet the criteria for accessing confidential data. Requests to access these datasets should be directed to faridaislahudin@ukm.edu.my.
