# Supplementary material for: Adverse Drug Reactions of Antihypertensives and CYP3A5*3 Polymorphism Among Chronic Kidney Disease Patients
Source: Front Pharmacol. 2022 Mar 14;13:848804. doi: 10.3389/fphar.2022.848804 (PMC8963814; doi:10.3389/fphar.2022.848804)
Supplement: Supplementary file 1 [file Table1.DOCX]

Supplementary Material

# Supplementary Table: Frequency of drug prescribed during study period, and number of adverse drug reactions for each drug by the number of patients prescribed.

| Type Of Medications^1^ | Number of patients, n (%) | Number of ADRs associated per patients prescribed, n (%) |
| --- | --- | --- |
| **Calcium Channel Blockers** |  |  |
| Amlodipine | 95 (47.5) | 6 (6.3) |
| Diltiazem | 12 (6.0) | - |
| Felodipine | 32 (16.0) | 1 (3.1) |
| Nifedipine | 2 (1.0) | - |
| Verapamil | 2 (1.0) | - |
| **ACE Inhibitors** |  |  |
| Captopril | 1 (0.5) | - |
| Enalapril | 11 (5.5) | - |
| Perindopril | 91 (45.5) | 9 (9.9) |
| Ramipril | 1 (0.5) | - |
| **Angiotensin II Blockers** |  |  |
| Irbesartan | 19 (9.5) | - |
| Losartan | 49 (24.5) | 4 (8.2) |
| Telmisartan | 31 (15.5) | 5 (16.1) |
| Valsartan | 23 (11.5) | 2 (8.7) |
| **Beta-Blockers** |  |  |
| Atenolol | 35 (17.5) | 4 (11.4) |
| Bisoprolol | 41 (20.5) | 1 (2.4) |
| Carvedilol | 1 (0.5) | - |
| Labetalol | 2 (1.0) | - |
| Metoprolol | 32 (16.0) | 3 (9.4) |
| Propranolol | 1 (0.5) | - |
| **Alpha-blockers** |  |  |
| Prazosin | 32 (16.0) | 3 (9.4) |
| Terazosin | 7 (3.5) | - |
| **Low-ceiling diuretics** |  |  |
| Hydrochlorothiazide | 29 (14.5) | 1 (3.4) |
| Indapamide | 2 (1.0) | - |
| **Aldosterone antagonists and other potassium-sparing agents** |  |  |
| Amiloride | 2 (1.0) | - |
| Eplerenone | 1 (0.5) | - |
| Spironolactone | 26 (13.0) | 2 (7.7) |
| **Others** |  |  |
| Minoxidil | 4 (2.0) | 1 (25.0) |
| Moxonidine | 3 (1.5) | - |

^1^ Numbers may not sum to group totals as one patient could be prescribed with more than one agent from the same drug class during the three-year study period
